# Supplementary material for: Rethinking performance crises in professional soccer: German coaches’ insights into systemic vulnerabilities and escalating dynamics
Source: PLoS One. 2026 Feb 27;21(2):e0343985. doi: 10.1371/journal.pone.0343985 (PMC12948067; doi:10.1371/journal.pone.0343985)
Supplement: S2 File — (DOCX) [file pone.0343985.s002.docx]

# S2 File. An example of abductive reasoning in practice

To illustrate how abductive reasoning was applied in this study, the following example demonstrates how empirical anomalies prompted theoretical reflection and refinement. According to the framework proposed by Jekauc et al. [1], a performance crisis is described as a downward spiral that begins with negative affective states following unmet performance expectations. These affective states trigger a cascade of intrapersonal (e.g., rumination, declining self-confidence) and interpersonal (e.g., conflicts, communication breakdowns) processes that mutually reinforce one another. However, several interview excerpts highlighted real-world complexities that challenge and extend the original framework:

“The reasons for a crisis can be manifold. There are so many different ones that we couldn’t possibly name them all.” (Coach 11)

This statement points to a range of causes for crises, rather than a single starting point (such as unmet expectations). It suggests that organizational, psychological, and contextual factors can all play a role, supporting the need for a broader view.

“Results do not always have to be the origin of a crisis; it can also happen the other way around. You can have an internal mood crisis that eventually leads to a results crisis.” (Coach 10)

This perspective reverses the direction assumed by the original model. It implies that internal team dynamics or mood disorders may drive poor results, which means that causality is more complex and can flow both ways.

“That’s why a results crisis is always a creeping process, because you always think after losing one game, ‘it’s just a single incident,’ and only after a series of defeats do you realize it’s a crisis.” (Coach 9)

Instead of a clear, cyclical progression, this statement describes a gradual and sometimes hidden development of crisis over time, questioning the notion of distinct crisis phases.

These insights led to important revisions of the cyclical crisis model proposed by Jekauc et al. [1]. The findings recognize a wide array of *pre-crisis vulnerabilities*, including external, club-internal, and team-internal risk factors, which can overlap and serve as different entry points into crisis. Therefore, the findings suggests that crises reflect reciprocal and nonlinear dynamics, where internal states and team processes may both precede and follow negative results. Finally, the findings suggest that crisis escalation can be a slow, extended process influenced by complex interactions among team and organizational dynamics.

In summary, by integrating these interview findings, the findings offer a flexible and comprehensive understanding of how crises emerge and develop in professional soccer teams, reflecting the nuanced reality captured through abductive analysis.

1. Jekauc D, Vrancic D, Fritsch J. Insights from elite soccer players: understanding the downward spiral and the complex dynamics of crises. Ger J Exerc Sport Res. 2024;54:429–41.
