# Supplementary material for: Rethinking performance crises in professional soccer: German coaches’ insights into systemic vulnerabilities and escalating dynamics
Source: PLoS One. 2026 Feb 27;21(2):e0343985. doi: 10.1371/journal.pone.0343985 (PMC12948067; doi:10.1371/journal.pone.0343985)
Supplement: S3 Table — Note. Translation from German. (DOCX) [file pone.0343985.s003.docx]

# S3 Table

**Table 2. Interview Guide**

| **Content** | **Key question/narrative prompt** | **Specific inquiries** |
| --- | --- | --- |
| **Part 1: Ice breaker / familiarization** | | |
| Familiarization / experience | First, I would like to ask you to tell me about your soccer coaching career. What stages have you gone through? |  |
| **Part 2: Definition of a performance crisis** | | |
| Experience and definition of a performance crisis by the interviewee | Have you ever experienced a performance crisis with a soccer team? If so, how would you define it? | *If the question arises about what a performance crisis is, you can provide the definition: You’re familiar with the phenomenon when a team consistently underperforms well below its potential over several consecutive matches.* |
| **Part 3: Personal experience with a performance crisis** | | |
| Narrative prompt / Crisis description | Could you describe the performance crisis you experienced in all its facets? | *What were the underlying mechanisms that led to the crisis?*  *Was there a specific moment when you realized that you were in a crisis?*  *What factors contributed to sustaining this crisis?*  *In your view, what contextual factors were crucial in either perpetuating or initiating the crisis?*  *What were the consequences of this crisis for the team?*  *What impact did this crisis have on the club?*  *How did this crisis personally affect you?* |
| Individual role and significance | What role did you adopt during the crisis?  What significance did this crisis hold for you personally? | *In your opinion, what is the coach’s contribution to the crisis?* |
| Individual intervention strategy | What strategies did you use to end the crisis?  How successful was this strategy?  From today's perspective, which strategies do you consider successful or unsuccessful? | *What role did you play in managing the crisis?*  *Do you have a specific philosophy or approach during such times of crisis?*  *Could you explain your philosophy or approach during these times of crisis?*  *How did you implement or apply this philosophy or strategy?* |
| Influence of the club/team on the resolution of the crisis | What contributions did the club officials and the team make towards resolving the crisis? | *Which club officials, in your opinion, were particularly crucial in resolving the crisis?*  *Why do you consider these individuals to be particularly important?* |
| **Part 4: Description of the relationships between the components of the crisis** | | |
| Facets of a crisis | You've previously listed some underlying mechanisms, sustaining factors, and consequences of your own crisis. Do you think there could be additional factors that you also consider relevant based on your experiences in soccer? | *Are there additional contextual factors/consequences/underlying mechanisms that could be crucial?* |
| Explanation of a crisis from the subject’s perspective | Could you explain how these components of the crisis are connected? | *How do you think a crisis can emerge?*  *How does a crisis develop over time?*  *What factors contribute to such a crisis?* |
| Intervention strategies in a crisis | With your current experience, how would you deal with such crises today? | *If different: Could you tell me why you would now choose a different strategy or philosophy?*  *If the same: Do you think there could be additional intervention strategies that could be significant?* |
| **Part 5: Closing question** | | |
| Open-ended closing question | That’s it from my side. We’ve discussed a few things now. Is there anything from your end that hasn’t been addressed in this interview but you feel is important in this context? Thank you for your time and openness in discussing this topic! |  |

Note. Translation from German
