# Supplementary material for: Rethinking performance crises in professional soccer: German coaches’ insights into systemic vulnerabilities and escalating dynamics
Source: PLoS One. 2026 Feb 27;21(2):e0343985. doi: 10.1371/journal.pone.0343985 (PMC12948067; doi:10.1371/journal.pone.0343985)
Supplement: S4 File — (DOCX) [file pone.0343985.s004.docx]

# S4 File. Detailed description of the data analysis process

The analysis followed the five-phase model proposed by Kuckartz [1]. In phase 1, the first author (CR) and a research assistant engaged in an initial familiarization with the data, which involved thorough reading of the interview transcripts and the writing of analytical memos. In phase 2, both CR and the research assistant independently developed a coding framework based on the theoretical framework of performance crises by Jekauc et al. (2024). After iterative discussions and reaching consensus, the framework was applied to all interviews in phase 3, which marked the first round of coding. During this phase, codes were generated in a concept-driven manner. For instance, in response to the question “What contributed to the performance crisis?”, a coach’s statement such as “At the very beginning, of course, the results” was categorized under *negative results*. In phase 4, a second round of coding was conducted to capture more nuanced, data-driven insights. This phase involved the development of new subcategories that were not previously included in the initial coding framework, reflecting novel themes emerging directly from the data. For example, the statement “But of course, there are also factors that lead to these negative results” represented a previously unrecognized concept and was accordingly coded as a new subcategory of *pre-crisis vulnerabilities*. This approach allowed for a more comprehensive representation of the coaches’ lived experiences and the dynamic complexity of performance crises*.* Finally, in phase 5, the results were documented in detail, and the analytical process was transparently described to ensure methodological rigor, traceability, and trustworthiness.

1. Kuckartz U. Qualitative Text Analysis: A Systematic Approach. In: Kaiser G, Presmeg N, editors. Compendium for Early Career Researchers in Mathematics Education. Cham: Springer International Publishing; 2019. p. 181-97.
