# Supplementary material for: Rethinking performance crises in professional soccer: German coaches’ insights into systemic vulnerabilities and escalating dynamics
Source: PLoS One. 2026 Feb 27;21(2):e0343985. doi: 10.1371/journal.pone.0343985 (PMC12948067; doi:10.1371/journal.pone.0343985)
Supplement: Attachment — Submitted filename: PONE-D-25-63850_reviewer_comments.docx [file pone.0343985.s005.docx]

Manuscript ID: PONE-D-25-53850

“Rethinking performance crises in professional 30 soccer: Coaches’ insights into systemic vulnerabilities and escalating dynamics”

A. **Summary of the Research and Overall Impression**

Thank you for the opportunity to review this paper. This manuscript presents a timely and conceptually rich qualitative exploration of how professional soccer coaches perceive the development and maintenance of performance crises. Anchored in an abductive, theory-informed thematic content analysis, the study advances current knowledge by integrating organizational, interpersonal, and psychological perspectives into a multi-level framework of crisis development. The explicit focus on the coaches’ standpoint, which has been comparatively underrepresented in the existing literature, constitutes a notable contribution.

Overall, the manuscript is theoretically well grounded, methodologically coherent, and empirically insightful. The integration of complexity theory, ecological systems theory, and sensemaking perspectives yields a nuanced understanding of how pre-crisis vulnerabilities, acute triggers, and escalating dynamics interact over time. At the same time, certain aspects of the presentation, particularly the density and layering of the findings section, the positioning of the study within the broader exploratory research tradition, and some minor issues in the reference formatting, would benefit from refinement. With revisions addressing these points, I believe the paper has strong potential to make a meaningful contribution to the literature on performance crises in professional sport.

**Strengths**

1. Provides an original and much‑needed focus on professional coaches’ perspectives, complementing player‑centred research on performance crises.
2. Develops a multi‑level, theoretically informed framework that links pre‑crisis vulnerabilities, crisis triggers, and escalating dynamics across organizational, team, and individual levels.
3. Offers practically relevant implications for organizational development, leadership alignment, and resilience-building in professional football.

**Weakness**

1. The Introduction could more explicitly integrate the key conceptual and theoretical frameworks (e.g., ecological systems, complexity in team dynamics) to create stronger continuity between the literature review, and the findings/discussion/conclusion.
2. The findings/results section is at times overly complex, with multiple nested layers and partially inconsistent terminology, which may challenge reader comprehensibility
3. The manuscript could position its exploratory qualitative design more explicitly within established methodological literature and clarify the use (or non-use) of formal intercoder agreement indices.
4. Minor but systematic issues in reference formatting (Vancouver style, capitalization, consistency across source types, internet references) require careful revision.

**Recommendation**
Overall, the manuscript makes a valuable contribution to the field and is suitable for publication after minor revisions.

B. **Discussion of Specific Areas for Improvement**

**Major Issues**

No major issues have been identified.

**Minor Issues**

- **INTRODUCTION:** In my view, a separate “Theoretical framework” section is not strictly necessary. However, it might be beneficial to enrich the latter part of the Introduction so that the main conceptual and theoretical underpinnings of the study are articulated more explicitly. For instance, the process-oriented models of performance crises, together with the ecological systems perspective, complexity theory, and sensemaking approach that are later used in the Discussion, could be more clearly introduced and integrated in the literature section. Such an expansion would not require adding an entirely new section, but rather refining the existing Introduction to ensure stronger conceptual continuity between the literature review, the findings, and the subsequent discussion. This would enhance the overall coherence of the manuscript and align it more closely with conventions in qualitative research reporting.
- The text convincingly establishes the aim and relevance of the study, particularly by demonstrating that the coach’s perspective has been largely neglected in previous research. A noteworthy point, however, appears in lines 110-114, where it is stated that clubs often enter cycles of failure without sufficiently learning from past crises and frequently resort to coach dismissals as the primary solution. I find this observation well grounded, yet I believe that briefly addressing the possible economic and sociological dimensions of this tendency could add further depth to the manuscript.
- For instance, designating the coaching staff as the “scapegoat” may represent a less costly and more “secure” option, both economically and symbolically, than confronting a squad of 22-30 players (lines 716-717 may perhaps offer a subtle hint here). Since fan identification and emotional attachment are largely formed through players, dismissing the coach rather than sanctioning or replacing allegedly problematic players may be perceived as a less controversial move in the eyes of supporters and the media. Moreover, when players are conceptualized as financial assets-linked to transfer fees, bonuses, and broadcasting-related revenues, clubs may consider it strategically more rational to replace the comparatively less protected coach than to “kill the goose that lays the golden eggs.” In addition to the financial burden of terminating contracts, asymmetric power relations may also arise from the potential legal disputes and further crises that could follow as stated at lines 636-638. (with the possible exception of very influential agents or highly established head coaches) I believe that briefly discussing these economic and sociological dynamics in the Discussion section could help to explain more comprehensively the decision logic underlying coach changes during performance crises. The treatment of this point in the Discussion section (lines 303–308) appears somewhat superficial. Expanding on this aspect with deeper conceptual or empirical insights would, in my view, substantially enhance the interpretive depth and overall clarity of the study.
- **METHODS:** The methods section is generally clear, systematic, and provides an adequate level of detail for a qualitative exploratory research design employing thematic content analysis. The integration of data-driven and concept-driven analytic approaches within an abductive (intuitive–deductive) logic offers a well-suited framework for exploring coaches’ perceptions of performance crises, consistent with the theoretical framework established by Jekauc et al. (2024).
- The application of the SRQR checklist, together with careful attention to trustworthiness criteria (Elo et al., 2014) and consensus coding procedures, demonstrates methodological rigor appropriate for exploratory qualitative designs. However, to further strengthen the methodological positioning, it would be beneficial for the authors to situate their abductive thematic content analysis more explicitly within the broader exploratory research tradition. For instance, brief references to foundational sources such as Lederman (1993) or Stebbins (2001) could help anchor the study’s methodological approach more firmly within the established exploratory research literature. While the reference to Rausch et al. (2025) provides valuable contextualization of the research group’s philosophical orientation (ontological relativism/constructivist epistemology), linking the research design, data collection strategy, and analytic procedures more explicitly to widely recognized methodological cornerstones in the qualitative literature would, in my view, further enhance perceptions of rigor and better situate the study within the broader qualitative research tradition.
- In addition, although the manuscript clearly describes the use of consensus coding, it remains unclear whether any formal index of intercoder agreement (e.g., Cohen’s kappa or a similar statistic) was calculated. Clarifying whether such an index was computed, and, if so, reporting its value, would further strengthen the transparency and trustworthiness of the analytic procedures.
- **RESULTS:** First of all, as this is a qualitative study, I would kindly suggest using the term “Findings” instead of “Results” for the corresponding section heading. In qualitative research, “findings” is more commonly used to emphasize the interpretive, meaning-oriented nature of the analysis, whereas “results” is more closely associated with quantitative, statistically driven outputs. This is, of course, only a recommendation, and I leave it to the authors’ discretion whether they consider this change appropriate for their manuscript.
- Figure 1 presents the overall model; however, the structure described in the text appears highly complex, with multiple nested layers. For example, under *Pre-crisis vulnerabilities*, there are *external vulnerabilities*, followed by subcategories such as *public expectations*, each of which is then elaborated with detailed explanations. I fully understand and appreciate your effort to present the richness of the findings. Considering that the journal’s publication format does not use numbered headings, the current structure contains too many nested layers and inconsistent naming conventions, making it challenging for readers to follow. Would it perhaps be sufficient to outline the general framework of the model and refer briefly to the main categories instead of providing such an extensive level of detail? I offer this as a suggestion, and I look forward to reading your reflections on this point in your response doc.
- I would like to highlight the finding presented in lines 491-506 as one of the most striking and insightful parts of the manuscript. Including a brief reference to this key result in the abstract could further strengthen the overall impact of the study and enhance its citation potential.
- **DISCUSSION:** In lines 763-770, the manuscript touches on what I consider one of its most original and compelling contributions. In some cases, attempts to resolve a problem are delegated to the very actors who contributed to its emergence, which can result in ineffective efforts and a vicious cycle of unproductive work. Within the football industry in particular, such unsuccessful interventions may not only cause psychological strain among fans but also lead to tangible and intangible losses for other stakeholders, including club employees at various levels, sponsors, and associated partners.
- It should be remembered that, regardless of whether football clubs are publicly supported or privately funded, they operate as legal entities in the contemporary sports industry with an influence that often exceeds their physical size or local base. Consequently, their failures can function like a “black hole,” drawing in and adversely affecting those around them. From this perspective, systematically identifying and diagnosing problems must be regarded as a crucial first step in any problem-solving process. All stakeholders, sometimes including the fans, need to demonstrate the willingness and capacity to assume responsibility when necessary. I would like to commend the authors for addressing these issues and for the considerable effort invested in this study.
- **Strengths and limitations:** In the “Strengths and limitations” section (line 855 onwards), the authors note that the exclusive focus on German football culture may limit the generalizability of the findings to other cultural contexts. However, it is well established that the primary aim of qualitative research is not statistical generalization, but rather the in-depth exploration and illumination of a given phenomenon within its specific context. From this perspective, the present study successfully uncovers a particular reality within the context of the German football tradition, but should not claim to explain structures and processes across the entire football industry. Instead of framing this as a limitation in terms of generalizability, it might be more consistent with the qualitative research design to emphasise how the findings and conclusions are interpreted in relation to the specific context under study. I would therefore kindly suggest that the authors explicitly foreground this contextual focus, either in the title and/or in the statement of the research aim in the introduction, so that the boundaries of the study are clearly delineated for the reader.
- **REFERENCES:** I would kindly recommend revising the reference list to ensure full compliance with the Vancouver style used by PLOS ONE. There appear to be some minor inconsistencies and deviations in the formatting of internet sources, the initial letters of journal names should be capitalized, and the same type of source (e.g., books) is presented in different formats and some more…
