# Supplementary material for: Rethinking performance crises in professional soccer: German coaches’ insights into systemic vulnerabilities and escalating dynamics
Source: PLoS One. 2026 Feb 27;21(2):e0343985. doi: 10.1371/journal.pone.0343985 (PMC12948067; doi:10.1371/journal.pone.0343985)
Supplement: Attachment — Submitted filename: Response To Reviewers.docx [file pone.0343985.s006.docx]

| **Review Comments** | |
| --- | --- |
| **Comment** | **Response** |
| **Reviewer #1** | |
| Thank you for the opportunity to review this paper. This manuscript presents a timely and conceptually rich qualitative exploration of how professional soccer coaches perceive the development and maintenance of performance crises. Anchored in an abductive, theory-informed thematic content analysis, the study advances current knowledge by integrating organizational, interpersonal, and psychological perspectives into a multi-level framework of crisis development. The explicit focus on the coaches’ standpoint, which has been comparatively underrepresented in the existing literature, constitutes a notable contribution. Overall, the manuscript is theoretically well grounded, methodologically coherent, and empirically insightful. The integration of complexity theory, ecological systems theory, and sensemaking 6 perspectives yields a nuanced understanding of how pre-crisis vulnerabilities, acute triggers, and escalating dynamics interact over time. At the same time, certain aspects of the presentation, particularly the density and layering of the findings section, the positioning of the study within the broader exploratory research tradition, and some minor issues in the reference formatting, would benefit from refinement. With revisions addressing these points, I believe the paper has strong potential to make a meaningful contribution to the literature on performance crises in professional sport. | We sincerely thank the reviewer for the thoughtful and encouraging evaluation of our manuscript. We greatly appreciate the recognition of the study’s conceptual contribution and methodological coherence. The points raised regarding the presentation of the findings, the positioning of the study within the broader exploratory research tradition, and the reference formatting have been carefully considered. We address each of these aspects in detail in the respective sections below. |
| Strengths   1. Provides an original and much-needed focus on professional coaches’ perspectives, complementing player-centred research on performance crises. 2. Develops a multi-level, theoretically informed framework that links pre-crisis vulnerabilities, crisis triggers, and escalating dynamics across organizational, team, and individual levels. 3. Offers practically relevant implications for organizational development, leadership alignment, and resilience-building in professional football. | We thank the reviewer for highlighting these strengths of the manuscript. We are pleased that the study’s focus on professional coaches, the development of a multi-level theoretical framework, and the practical implications for leadership and organizational development were recognized as valuable contributions. |
| Weakness   1. The Introduction could more explicitly integrate the key conceptual and theoretical frameworks (e.g., ecological systems, complexity in team dynamics) to create stronger continuity between the literature review, and the findings/discussion/conclusion. 2. The findings/results section is at times overly complex, with multiple nested layers and partially inconsistent terminology, which may challenge reader comprehensibility 3. The manuscript could position its exploratory qualitative design more explicitly within established methodological literature and clarify the use (or non-use) of formal intercoder agreement indices. 4. Minor but systematic issues in reference formatting (Vancouver style, capitalization, consistency across source types, internet references) require careful revision. | We thank the reviewer for these constructive and detailed suggestions. We fully agree that the aspects mentioned will further strengthen the clarity and coherence of the manuscript. Accordingly, we have revised the Introduction to more explicitly connect the key conceptual frameworks (ecological systems theory, complexity theory) with the subsequent findings and discussion.  We thank the reviewer for highlighting the need for improved readability and terminological consistency in the Findings section. To address this, we have streamlined the structure by reducing nesting depth (e.g., consolidating Level 4 subcategories under Level 3, now max 3 levels), and the exploratory qualitative design is now more clearly positioned within established methodological literature. We have also clarified our approach to intercoder agreement and ensured full consistency in reference formatting according to Vancouver style.  These revisions are described in detail in the respective sections below. |
| **Introduction** | |
| In my view, a separate “Theoretical framework” section is not strictly necessary. However, it might be beneficial to enrich the latter part of the Introduction so that the main conceptual and theoretical underpinnings of the study are articulated more explicitly. For instance, the process-oriented models of performance crises, together with the ecological systems perspective, complexity theory, and sensemaking approach that are later used in the Discussion, could be more clearly introduced and integrated in the literature section. Such an expansion would not require adding an entirely new section, but rather refining the existing Introduction to ensure stronger conceptual continuity between the literature review, the findings, and the subsequent discussion. This would enhance the overall coherence of the manuscript and align it more closely with conventions in qualitative research reporting. | Thank you for this constructive suggestion, which we fully agree enhances the manuscript’s conceptual coherence. To articulate the main theoretical underpinnings more explicitly without adding a separate section, we have refined the latter part of the Introduction (lines 100-114). Here, the process-oriented models of performance crises (Buenemann et al., 2023; Jekauc et al., 2024) are now integrated with the ecological systems perspective (Bronfenbrenner, 1979), key principles of complexity theory (e.g., interconnectedness, emergence, non-linear feedback; Balagué et al., 2013), and sensemaking theory (Weick, 1995, 2005). These frameworks are introduced as complementary perspectives that highlight multi-level dynamics, cascading effects, and interpretive challenges in crises, thereby ensuring greater continuity between the literature review, our findings on vulnerabilities/triggers/escalation, and the subsequent discussion.  The added paragraph reads (lines 100-114):  “These models of performance crises can be enriched by broader theoretical perspectives that emphasize multi-level interactions and interpretive processes. From an ecological systems viewpoint [9] and key principles of complexity theory, such as interconnectedness, emergence, and non-linear feedback [10], crises emerge from dynamic interdependencies across nested environments. Here, micro-level triggers can cascade into meso-level systemic threats, as articulated by Szymanski and Weimar [4], who identify deviations from expected team performance as key drivers of insolvency risk in professional soccer. Complementarily, sensemaking theory [11] describes a retrospective process in which individuals interpret ambiguous cues. Weick states, that individuals act ahead of cognition, “acting their way into belated understanding” [12 p419]. Within this framework, in times of performance crisis, a consensus may not be reached by club officials, coaches, and players due to divergent realities being experienced, exacerbated by mismatched performance expectations. These divergent realities could lead to breakdowns, such as coach dismissals and self-reinforcing instability, hindering learning and resolution [13].” |
| The text convincingly establishes the aim and relevance of the study, particularly by demonstrating that the coach’s perspective has been largely neglected in previous research. A noteworthy point, however, appears in lines 110-114, where it is stated that clubs often enter cycles of failure without sufficiently learning from past crises and frequently resort to coach dismissals as the primary 7 solution. I find this observation well grounded, yet I believe that briefly addressing the possible economic and sociological dimensions of this tendency could add further depth to the manuscript. | Thank you for this constructive feedback, which strengthens the manuscript’s theoretical depth. We have revised lines 118-126 in the Introduction to explicitly address the economic and sociological dimensions of coach dismissals, incorporating scapegoat theory alongside qualitative and empirical evidence from relevant studies.  **Key Revisions**   - Sociological dimensions: Added reference to Galdino et al. (2025), detailing social construction via media pressure, club politics, poor planning, illogical expectations, and player disengagement, explaining why coaches become scapegoats under asymmetric pressures. - Economic dimensions: Integrated empirical null effects from Flores et al. (2012; Argentine dismissals) and Heuer et al. (2011; Bundesliga), underscoring irrationality and cost-efficiency of coach changes over squad overhauls.   The added text reads (lines 118-126):  This tendency can be explained through the scapegoat theory, which posits that coaches serve as symbolic scapegoats to displace frustrations from stakeholders, without sustainably improving performance [15]. Galdino et al. [16] qualitatively explains these decisions through the social construction of leadership, driven by excessive media pressure, dominant politics in club administration, lack of strategic planning, illogical expectations, and player disengagement. Empirically, Flores et al. [17] analyzed dismissals of Argentine soccer coaches under pressure and found no performance gains. Similarly, Heuer et al. [18] demonstrated null effects in the German Bundesliga due to regression to the mean.  These additions enhance interpretive clarity while maintaining conciseness. We believe this fully addresses your suggestion and bolsters the study’s relevance. |
| For instance, designating the coaching staff as the “scapegoat” may represent a less costly and more “secure” option, both economically and symbolically, than confronting a squad of 22-30 players (lines 716-717 may perhaps offer a subtle hint here). Since fan identification and emotional attachment are largely formed through players, dismissing the coach rather than sanctioning or replacing allegedly problematic players may be perceived as a less controversial move in the eyes of supporters and the media. Moreover, when players are conceptualized as financial assets-linked to transfer fees, bonuses, and broadcasting-related revenues, clubs may consider it strategically more rational to replace the comparatively less protected coach than to “kill the goose that lays the golden eggs.” In addition to the financial burden of terminating contracts, asymmetric power relations may also arise from the potential legal disputes and further crises that could follow as stated at lines 636- 638. (with the possible exception of very influential agents or highly established head coaches) I believe that briefly discussing these economic and sociological dynamics in the Discussion section could help to explain more comprehensively the decision logic underlying coach changes during performance crises. The treatment of this point in the Discussion section (lines 303–308) appears somewhat superficial. Expanding on this aspect with deeper conceptual or empirical insights would, in my view, substantially enhance the interpretive depth and overall clarity of the study. | Thank you for this insightful suggestion, which precisely identifies opportunities to deepen the economic and sociological rationale for coach scapegoating. We fully agree that the discussion section appeared somewhat superficial and have therefore expanded the Discussion section to integrate these dynamics, drawing on conceptual and empirical sources that echo your points on costs, fan/media perceptions, player assets, and power asymmetries.  **Key Revisions**   - Economic aspects: Explicitly addressed lower costs/symbolic security of coach dismissal vs. squad (22-30 players), player-as-assets (transfers/bonuses/revenues), and ‘goose that lays golden eggs’ via balance sheet valuations and cost-per-point risks. - Sociological aspects: Incorporated fan identification, media/support perceptions, and legal disputes   The added text reads (lines 826-838):  From an ecological standpoint, this reactive decision-making is comprehensible, as scapegoating the coach is economically less costly and strategically more secure than confronting the squad. Players are reflected as intangible assets on balance sheets [48], drive broadcasting revenues through media value [49], and significantly enhance enterprise value [50], while coaches of higher-budget teams face elevated dismissal risks due to inefficient cost-per-point performance, reflecting their expendability relative to squad investments [51]. Especially “when the narrative demands a solution and the media chase in herds; when coaches become victim of the ‘media-hunt’” [52 p169], scapegoating avoids terminating high-value player contracts, potential legal disputes, and controversy with fans/media emotionally attached to players over staff. This aligns with organizational theory where scapegoating thrives amid attributional ambiguity, enabling blame-shifting to individuals while avoiding costly squad restructuring [53].  These enhancements provide deeper conceptual/empirical support, directly responding to your examples while tying back to our findings on vulnerabilities. The section now offers comprehensive decision logic for coach changes in crises, improving interpretive clarity. |
| **Methods** | |
| The methods section is generally clear, systematic, and provides an adequate level of detail for a qualitative exploratory research design employing thematic content analysis. The integration of data-driven and concept-driven analytic approaches within an abductive (intuitive– deductive) logic offers a well-suited framework for exploring coaches’ perceptions of performance crises, consistent with the theoretical framework established by Jekauc et al. (2024). | We appreciate the reviewer’s positive assessment of the Methods section. We are pleased that the description of our abductive thematic content analysis and the integration of data-driven and concept-driven approaches were found to be clear and consistent with the theoretical framework by Jekauc et al. (2024). |
| The application of the SRQR checklist, together with careful attention to trustworthiness criteria (Elo et al., 2014) and consensus coding procedures, demonstrates methodological rigor appropriate for exploratory qualitative designs. However, to further strengthen the methodological positioning, it would be beneficial for the authors to situate their abductive thematic content analysis more explicitly within the broader exploratory research tradition. For instance, brief references to foundational sources such as Lederman (1993) or Stebbins (2001) could help anchor the study’s methodological approach more firmly within the established exploratory research literature. While the reference to Rausch et al. (2025) provides valuable contextualization of the research group’s philosophical orientation (ontological relativism/constructivist epistemology), linking the research design, data collection strategy, and analytic procedures more explicitly to widely recognized methodological cornerstones in the qualitative literature would, in my view, further enhance perceptions of rigor and better situate the study within the broader qualitative research tradition. | Thank you for this constructive feedback on the methodological positioning. We greatly appreciate your recognition of the methodological rigor demonstrated through the SRQR checklist, trustworthiness criteria (Elo et al., 2014), and consensus coding procedures.  We also want to thank you for this insightful feedback and for the valuable literature suggestions, which help situate our study within a broader exploratory research tradition. We fully agree with your assessment and have revised the manuscript accordingly to more explicitly link our study to established methodological cornerstones in qualitative research.  To address your recommendation, we integrated Stebbins (2001) directly following the philosophical orientation (Rausch et al., 2025), framing our study as qualitative-exploratory research (see revised text in Methods section, lines 151-156):  Accordingly, this study is framed as qualitative, exploratory research, following Stebbins’s tradition [23]. This approach involves a broad, purposeful, and systematic method to maximize discovery. The goal is to gain a better understanding of an understudied social phenomenon. In this case, the discovery includes coaches’ perceptions of systemic vulnerabilities, triggers, and escalating dynamics in performance crises.  This anchors our design, data collection, and analytic procedures in established exploratory literature, emphasizing inductive generalization discovery for description and understanding, distinct from confirmatory approaches. |
| In addition, although the manuscript clearly describes the use of consensus coding, it remains unclear whether any formal index of intercoder agreement (e.g., Cohen’s kappa or a similar statistic) was calculated. Clarifying whether such an index was computed, and, if so, reporting its value, would further strengthen the transparency and trustworthiness of the analytic procedures. | We thank the reviewer for highlighting this important point regarding methodological transparency. In line with our constructivist epistemology and the qualitative research tradition employed, formal intercoder agreement indices such as Cohen’s kappa were not calculated, as these metrics are more appropriate for positivist designs assuming objective “truth” in coding rather than the interpretive, consensus-driven process central to our abductive thematic analysis.  To address this, we have revised the relevant section (lines 271-276) as follows:  In alignment with our ontological relativism and constructivist epistemology, intercoder reliability was achieved through consensus coding [32], which acknowledges the inseparability of the coding process from researchers’ subjectivities [33]. Accordingly, CR and a research assistant engaged in frequent critical dialogues and weekly meetings to reflexively discuss, examine, and resolve coding discrepancies until a shared understanding was achieved.  This revision explicitly states that no formal index of inter-coder agreement was calculated and explains our philosophically consistent rationale. This improves the transparency of our methodology without compromising the constructivist integrity of our analysis. |
| **Results** | |
| First of all, as this is a qualitative study, I would kindly suggest using the term “Findings” instead of “Results” for the corresponding section heading. In qualitative research, “findings” is more commonly used to emphasize the interpretive, meaning-oriented nature of the analysis, whereas “results” is more closely associated with quantitative, statistically driven outputs. This is, of course, only a recommendation, and I leave it to the authors’ discretion whether they consider this change appropriate for their manuscript. | We fully agree with the reviewer’s recommendation and have changed the section heading from “Results” to “Findings”. This adjustment better reflects the interpretive, meaning-oriented nature of our thematic content analysis. |
| Figure 1 presents the overall model; however, the structure described in the text appears highly complex, with multiple nested layers. For example, under *Pre-crisis vulnerabilities*, there are *external vulnerabilities*, followed by subcategories such as *public expectations*, each of which is then elaborated with detailed explanations. I fully understand and appreciate your effort to present the richness of the findings. Considering that the journal’s publication format does not use numbered headings, the current structure contains too many nested layers and inconsistent naming 8 conventions, making it challenging for readers to follow. Would it perhaps be sufficient to outline the general framework of the model and refer briefly to the main categories instead of providing such an extensive level of detail? I offer this as a suggestion, and I look forward to reading your reflections on this point in your response doc. | We appreciate the reviewer’s thoughtful suggestion to outline only the general framework with brief references to main categories. We fully understand and share your concern that the extensive subcategories (e.g., under pre-crisis vulnerabilities) could appear overly complex and risk overwhelming readers, particularly with four levels of nesting inconsistent with journal style.  However, the subcategories (e.g., diversity of perspectives, organizational incongruence) under the Level 3 headings (e.g., club-internal vulnerabilities) form the core narrative explaining mechanisms of crisis development and maintenance, illustrating how latent tensions interact and escalate across levels. This granularity reveals multi-layered, self-reinforcing processes fundamental for understanding key insights like “fixes that fail” (Senge, 1990) or sensemaking theory (Weick, 1995). This depth is particularly crucial to enable a comprehensive Discussion linking findings to economic/sociological dynamics of coach dismissals (e.g., scapegoating coaches as “less protected” targets vs. “golden egg” players), which requires precise mapping of vulnerabilities/escalations to explain decision logic beyond superficial description.  We acknowledge the reviewer’s point regarding PLOS’s non-numbered heading style and acknowledge that four levels of headings would indeed be problematic. Accordingly, we have restructured the text so that subcategories now appear as integrated elements within the Level 3 heading paragraphs rather than as a separate Level 4 headings. This maintains analytical depth while ensuring clarity and structural simplicity. |
| • I would like to highlight the finding presented in lines 491-506 as one of the most striking and insightful parts of the manuscript. Including a brief reference to this key result in the abstract could further strengthen the overall impact of the study and enhance its citation potential. | We thank the reviewer for highlighting this key finding as particularly insightful. Following this valuable suggestion, we have incorporated a reference to it, emphasizing how success temporarily masks pre-crisis vulnerabilities that surface during performance decline, into the abstract (now 297 words, not exceeding 300; line 38/lines 41-43). |
| **Discussion** | |
| In lines 763-770, the manuscript touches on what I consider one of its most original and compelling contributions. In some cases, attempts to resolve a problem are delegated to the very actors who contributed to its emergence, which can result in ineffective efforts and a vicious cycle of unproductive work. Within the football industry in particular, such unsuccessful interventions may not only cause psychological strain among fans but also lead to tangible and intangible losses for other stakeholders, including club employees at various levels, sponsors, and associated partners. | We sincerely thank the reviewer for highlighting this point as one of the manuscript’s most original and compelling contributions. We fully agree that the dynamic described represents a core insight of the study. In response, we have slightly expanded this section to clarify how problem-solving is often delegated to the same actors (e.g., coaches) who contributed to the vulnerabilities, thereby perpetuating ineffective interventions and associated consequences for various stakeholders.  Added text (lines 781-785):  Notably, this dynamic often results in problem-solving being delegated to the same actors (e.g., coaches) who contributed to the vulnerabilities, perpetuating ineffective interventions and vicious cycles that impose psychological strain on fans alongside tangible/intangible losses for stakeholders like employees, sponsors, and partners. |
| It should be remembered that, regardless of whether football clubs are publicly supported or privately funded, they operate as legal entities in the contemporary sports industry with an influence that often exceeds their physical size or local base. Consequently, their failures can function like a “black hole,” drawing in and adversely affecting those around them. From this perspective, systematically identifying and diagnosing problems must be regarded as a crucial first step in any problem-solving process. All stakeholders, sometimes including the fans, need to demonstrate the willingness and capacity to assume responsibility when necessary. I would like to commend the authors for addressing these issues and for the considerable effort invested in this study. | Thank you for this thoughtful observation and for your recognition of our work. We appreciate your framing of soccer clubs as influential organizations whose crises can extend beyond their immediate boundaries. To reflect this idea, we have revised the Implications subsection to emphasize the systemic repercussions of club crises and the importance of shared stakeholder responsibility in mitigating their effects.  Added text (lines 859-864):  Thus, systematically identifying and diagnosing pre-crisis vulnerabilities at all levels is a crucial first step. In the interconnected sports ecosystem, crises affecting professional soccer clubs can strongly impact surrounding stakeholders, including fans, employees, and sponsors, thereby amplifying their consequences. This interdependence highlights the importance of shared responsibility in strengthening collective resilience and preventing cascading failures [4].  This addition honors your perspective while tying seamlessly to our multi-level resilience building, enhancing practical relevance. |
| Strengths and limitations: In the “Strengths and limitations” section (line 855 onwards), the authors note that the exclusive focus on German football culture may limit the generalizability of the findings to other cultural contexts. However, it is well established that the primary aim of qualitative research is not statistical generalization, but rather the in-depth exploration and illumination of a given phenomenon within its specific context. From this perspective, the present study successfully uncovers a particular reality within the context of the German football tradition, but should not claim to explain structures and processes across the entire football industry. Instead of framing this as a limitation in terms of generalizability, it might be more consistent with the qualitative research design to emphasise how the findings and conclusions are interpreted in relation to the specific context under study. I would therefore kindly suggest that the authors explicitly foreground this contextual focus, either in the title and/or in the statement of the research aim in the introduction, so that the boundaries of the study are clearly delineated for the reader. | We greatly appreciate the reviewer’s insightful feedback on the qualitative nature of our study and the emphasis on contextual specificity, which aligns perfectly with our methodological approach. Following this suggestion, we have reframed the “Strengths and limitations” section to highlight the contextual focus as a strength of the in-depth exploration within German professional soccer.  Added text (lines 897-900):  Third, the bounded focus on male German professional soccer coaches intentionally illuminates context-specific norms and structures, prioritizing qualitative transferability over statistical generalizability to other cultures [37].  Additionally, we have explicitly delineated the study boundaries by adjusting the title to “Rethinking performance crises in professional soccer: German coaches’ insights into systemic vulnerabilities and escalating dynamics”, and by refining the research aim in the introduction to stress the examination within German football traditions (lines 142-143):  “within the specific context of German male professional soccer.”  Finally, we expanded the “Future research” section to propose comparative studies in other national soccer cultures, gender roles, and sports, underscoring potential contextual differences, thereby highlighting possible contextual differences (lines 924-929):  Lastly, for a deeper understanding of the performance crisis and how it differs in various contexts, it would be useful to conduct similar studies in other types of sports as well as in women’s professional soccer and other national soccer cultures, where differing norms around leadership, fan expectations, and organizational structures may shape distinct crisis dynamics [63].  These changes strengthen the manuscript’s clarity and consistency with qualitative principles. |
| **References** | |
| I would kindly recommend revising the reference list to ensure full compliance with the Vancouver style used by PLOS ONE. There appear to be some minor inconsistencies and deviations in the formatting of internet sources, the initial letters of journal names should be capitalized, and the same type of source (e.g., books) is presented in different formats and some more… | We appreciate the reviewer’s attention to detail in identifying these formatting inconsistencies. The reference list has been revised to adhere fully to the Vancouver guidelines of PLOS ONE, with standardized journal capitalization and a uniform presentation of source types (e.g., books). The formatting of online resources has also been corrected. |
| **Reviewer #2** | |
| I would like to thank the editors for the opportunity to review the manuscript entitled “Rethinking performance crises in professional soccer: Coaches’ insights into systemic vulnerabilities and escalating dynamics.” I also congratulate the authors for addressing a timely and relevant topic within the field of professional sport, offering an in-depth and thoughtful analysis of performance crises from the perspective of coaches.  Overall, the manuscript is well written and presents a clear and coherent structure. The rationale is well articulated, the research question is logically derived from the literature, and the methodological approach is appropriate and consistent with the study’s objectives. The alignment between the theoretical framework, data collection, and analysis strengthens the credibility and contribution of the work. | We sincerely thank the reviewer for this positive assessment and encouragement. Your recognition of the manuscript’s structure, rationale, and methodological rigor is greatly appreciated and motivates us to further refine the work. |
| That said, I would like to raise just one point for clarification. It is not clear whether the study refers to professional men’s soccer, women’s soccer, or both. This ambiguity appears both in the title and in the methods section, where the gender of the sport context and of the participants (coaches) is not explicitly stated. This information is highly relevant, as the structural conditions, organizational contexts, and potential sources of performance crises may differ substantially between men’s and women’s professional soccer.  Clarifying whether the study focuses on men’s soccer, women’s soccer, or a mixed context—and specifying the gender of the participants—would significantly strengthen the manuscript, improve transparency, and enhance the interpretation and transferability of the findings. | We thank the reviewer for this perceptive comment, which improves the manuscript’s transparency. The study exclusively examines professional men’s soccer in Germany. We have clarified this in the methods section under “Sampling and participants” by adding: “All participants were male coaches from German professional men’s soccer leagues” (now lines 193-194). This specification resolves the ambiguity and supports context-specific interpretation of the findings. Relatedly, we have expanded the “Future research” section to explicitly suggest studies in women’s professional soccer alongside other contexts (lines 924-929):  Lastly, for a deeper understanding of the performance crisis and how it differs in various contexts, it would be useful to conduct similar studies in other types of sports as well as in women’s professional soccer and other national soccer cultures, where differing norms around leadership, fan expectations, and organizational structures may shape distinct crisis dynamics [63]. |
